# Pulpless Teeth Also Subject to Erosion

**Published:** 1893-11

**Authors:** J. M. Howe


ARTICLE III.

### PULPLESS TEETH ALSO SUBJECT TO EROSION.

BY J. M. HOWE.

In 1881, in the discussion of a paper read before the American Dental Association, Dr. Litch said that erosion unquestionably required "a vital condition of the tooth-structure for its development," and that he had never seen it occur in a devitalized tooth. And Dr. G. H. Cushing corroborated the statement as to the immunity of such teeth.

In January, 1884, in the discussion of a paper by Dr. E. E. Darby, Dr. Guilford said that when pulpless teeth are found eroded, "it has probably taken place before the teeth lost their vitality," Dr. Darby said that he had one case of erosion in a pulpless tooth, but the next year,—1885,—the same subject being under discussion, Dr. Darby said that he was not sure whether the erosion occurred before devitalization or afterwards.

But the other and more noticeable point in these quotations is the constantly recurring idea that the vitality of the dental tissues was in some way a factor in the destructive process, and a frequent expression of doubt whether pulpless teeth were subject to erosion. I fell into this error myself several years ago, and proposed that the nutrition of the teeth was probably concerned in the disease.

And although Dr. Miller's experiment—in which he obtained eroding action on ivory in the mouth—was relevant, it was hardly to be regarded as conclusive. It was, therefore, with unusual interest that I observed in the mouth of Mrs. N——, in February, 1892, the erosion of a pulpless central incisor, together with similar effects on several other incisors having living pulps.

I had seen this lady's teeth ten months previously, and knew that there was no erosion. There was a history, meantime, of vital depression, with general hyperæsthesia, and in the latter the teeth sympathized. Again, within the last two weeks, I have seen on the teeth of Mrs. H—— the beginning of erosion on the superior left central,—with a vital pulp,—and also on the pulpless right lateral. There has never been any gingival recession or erosion in this lady's mouth, but both of these lesions have affected these two teeth within the past year under my personal observation.

The observation made in these two cases that pulpless teeth are subject to erosion came to me as the revelation of a fact that I had not been aware of, and which the records show has been the subject of much doubt with others; but further back I have found it stated, in Maury's Dental Surgery,—1843,—that Dr. E. Parmly had recorded an observation as setting this question "beyond all doubt," that "a gentleman who had lost his teeth, partly from this cause came to us about four years ago . . . to procure artificial substitutes. A set of human teeth of the best quality were accordingly provided, which, in the course of three years and a half, on his return to us, were found to be grooved from the surface to the central cavity; and this same reference to Dr. Parmly's record is made to-day in Harris' "Principles and Practice." So that this fact,—that pulpless teeth are subject to erosion,—which has been a point of considerable speculation and assumption for years, and which I supposed I had made an original observation upon, was quite fully settled by Dr. Parmly's observation of fifty years ago. We may positively exclude, then, all further consideration of the vitality of the eroded tissue, or of its nutrition, as being a factor in its destruction.—*International Dental Journal.*

